# Croup

**Published:** 1861-08

**Authors:** 


**CROUP** is an inflammation of the inner surface of the windpipe. Inflammation implies heat, and that heat must be subdued or the patient will inevitably die. If prompt efforts are made to cool the parts in case of an attack of croup, relief will be as prompt as it is surprising and delightful. All know that cold applied to a hot skin cools it, but all do not as well know and understand, that hot water applied to an inflamed skin will as certainly cool it off. Hence the application of ice-cold water with linen cloths, or of almost boiling water with woolen flannel, are very efficient in the cure of croup. Take two or three pieces of woolen flannel of two folds, large enough to cover the whole throat and upper part of the chest, put these in a pan of water as hot as the hand can bear, and keep it thus hot, by adding water from a boiling tea-kettle at hand; let two of the flannels be in the hot water all the time, and one on the throat all the time, with a dry flannel covering the wet one, so as to keep the steam in to some extent; the flannels should not be so wet, when put on, as to dribble the water, for it is important to keep the clothing as dry as possible, and the body and feet of the child comfortable and warm. As soon as one flannel gets a little cool, put on another hot one, with as little interval of exposure as possible, and keep up this process until the doctor comes, or until the phlegm is loose, the child easier, and begins to fall to sleep; then gently wrap a dry flannel over the wet one which is on, so as to cover it up thoroughly, and the child is saved. When it wakes up, both flannels will be dry. The same results will follow if cold water is used, the colder the better; the cloths should be of muslin or linen and of several folds thickness, large enough to cover the whole throat and the upper part of the breast. Hold a dry flannel over the wet muslin, and as soon as the latter gets a very little warm, replace it with an ice-cold one. In this manner the most remarkable and grateful relief will be experienced within fifteen minutes, if the false membrane has not begun to form. If these applications are commenced on the first approach of croupy symptoms, they may be necessary for an hour or two; if delayed until death is imminent, they should be continued for days, if necessary; easier breathing, looser phlegm, and lessening restlessness being the certain signs of improvement, that danger is passing away.

Thus hot and cold water are both efficacious, because heat is essential to inflammation; if the heat is diminished, the inflammation must subside; cold water applied to the skin with successive ice-cold cloths, or a stream of cold water, or by a cake of snow, or a piece or bag of ice, causes great coldness in a direct manner. Hot water cools the surface by becoming steam, which absorbs an immense amount of heat, and rising, carries it from the body. Water absorbs more than a thousand degrees of heat before it becomes steam or vapor. Hot water is a more agreeable, a less shocking method of affording relief than cold, and is, perhaps, more generally accessible than ice-cold water. Linen cloths should be used with cold water, because it is known that a piece of wet linen or muslin applied to the skin feels colder than a piece of wet flannel, both having been dipped in cold water. The linen is a better conductor, carries off heat more rapidly. In the use of hot water, on the other hand, woolen should be used, because the object is to increase the heat, or rather retain it so as the more speedily to convert the water into steam, which carries off heat with many times greater rapidity than an ice-cold linen cloth. The reasons of these things are mentioned so that their application may be made more understandingly, and consequently more efficiently. Croup is a disease of early childhood, and is always brought on by a cold, generally resulting from exposure to the raw, damp air of sundown in the early spring and later fall, March and November.

The grandest, happiest hour of Jacob's life did not seem thus grand and happy, until it had departed, not to be repeated thus forever. And so are present hours of sunshine to us ever passing, but we recognize the sunshine only by the coming shade. Happy they who are wise enough to perceive and feel and enjoy life's sunshines while they are present. There are many who not only have not enjoyed their sunshine, were not conscious of its presence, but when it has passed, make life miserable in the vain regrets of not having appreciated and improved it while it was passing.

What heart-pangs daily seize on many a child (now grown to manhood) in memory of days departed, when father and mother were alive, in that they had not done more to make that father and mother happy; in that they had not done more to consult their wishes, to soothe their sorrows, to smooth their pillows, and strew their later pathway with flowers and smiles and sweet caresses; how sadly, and as vain as sad, do they remember thousands of opportunities wherein by little attentions, costing nothing at the time worth naming, they might have moved a stone out of their way and put a rose where was a thorn, or sung a song where there was a sorrow.

Many a time does the heart-breaking apostrophe leap from the bosom prison: My sister, why was I not kinder to thee! Dear brother, why did I not lend you more of my sympathy, and by kindly counsel and more substantial aid, help you in the time of your discouragement and trouble! And quite as often in the married relation is there a failure of proper appreciation of the husband or wife, until beyond all sympathy or praise; then vain the apostrophe: Could I have you back but one hour, how would my inmost soul leap out in love and my eyes run rivers of penitential water!

To go down to the grave then without bitter remorses, to have an old age crowded with dear delightful memories, cultivate the habit of perceiving and enjoying the present sunshine, of appreciating present blessings and present happiness; cultivate, sedulously cultivate a respectful affectionate attention to parental wishes, to the promotion of parental comfort and peace and quietude and gladness, and to all your kindred, especially to those nearest to you; aim steadily, not merely to discharge your whole duty, for that is a cold word in this connection, but let your whole life go out to them in willing sympathies, in timely assistance, in generous allowances, and in forbearances loving, and long, and sweet. Such a course will bring present rewards, and will lay up for the future a store of delightful satisfactions to be feasted on till life's latest hour.

